# Temporal perception in visual processing as a research tool

**DOI:** 10.3389/fpsyg.2015.00521

**Published:** 2015-04-24

**Authors:** Bin Zhou, Ting Zhang, Lihua Mao

**Affiliations:** ^1^Key Laboratory of Mental Health, Institute of Psychology, Chinese Academy of Sciences, Beijing, China; ^2^Department of Psychology, Peking University, Beijing, China

**Keywords:** subjective time, perceptual organization, research tool, neural response, object perception

## Abstract

Accumulated evidence has shown that the subjective time in the sub-second range can be altered by different factors; some are related to stimulus features such as luminance contrast and spatial frequency, others are processes like perceptual grouping and contextual modulation. These findings indicate that temporal perception uses neural signals involved in non-temporal feature processes and that perceptual organization plays an important role in shaping the experience of elapsed time. We suggest that the temporal representation of objects can be treated as a feature of objects. This new concept implies that psychological time can serve as a tool to study the principles of neural codes in the perception of objects like “reaction time (RT).” Whereas “RT” usually reflects the state of transient signals crossing decision thresholds, “apparent time” in addition reveals the dynamics of sustained signals, thus providing complementary information of what has been obtained from “RT” studies.

## Introduction

One of the extensively studied temporal topics is duration or interval timing in the sub-second range, with which a basic experience beyond instantaneity can be obtained (Fraisse, 1984). Such temporal processing is distinguished from time estimation of supra-second intervals that involves higher-order cognitive strategies (Fraisse, 1984). Whereas perceptual signal integration across tens to hundreds milliseconds is assumed to be automatic and pre-semantic (Pöppel, 1997, 2009), the feeling of how long an event lasts on this timescale appears to engage some active cognitive processes such as memory operations. Apparent time and related phenomena are often retrospectively constructed, and the context in which the temporal pattern is embedded usually exerts a modulatory effect on the time that one experiences (Eagleman, 2008; Bao et al., 2013). This retrospective and context-sensitive characteristic has also been discussed by Zhou et al. (2014a) and may have some connections with previous opinions on the phenomenological feature of subjective time (Woodrow, 1951; Gibson, 1975). Given that the spatiotemporal context often determines how stimuli are perceptually organized and perceived (Kramer and Yantis, 1997; Wagemans et al., 2012), it is important to consider the perceptual organization and its relationship to subjective time when one tries to understand the mechanisms underpinning various temporal phenomena.

## Sub-Second Time is Modulated by Perceptual Organization

Traditionally, the temporal signature of an event is examined separately from the content of that event, with the assumption that time and object features are represented by independent neural substrates (e.g., Pöppel, 1997, 2009). This independence, however, may not be true, considering that temporal information is most likely decoded from neural signals which process object features. The spiking patterns and the signal-to-noise ratio of neural activities involved in object perception thus may have an influence on the temporal decoding processes. This argument is consistent with the view that the encoding of object-identity is one of the fundamental requirements to unify different temporal phenomena (Zhou et al., 2014a). Relevant opinions are also expressed as the hypothesis that neural coding efficiency offers a basis of subjective time (Eagleman and Pariyadath, 2009). More detailed models suggest that subjective time is the output of multi-stage processes with context dependent circuits interacting with the core temporal machinery (Merchant et al., 2013). In our view, the context dependent stages include object perception processes and provide feeding signals for the computation that may function at the central stage. What is perceptually represented (i.e., the content) may alter the interaction and thus modulate how the signals are decoded.

Previous studies have accumulated evidence supporting the association of object processes and perceived time; for example, apparent duration of a visual stimulus is modulated by a preceding prime stimulus (Zhou et al., 2010). When the stimulus-onset-asynchrony (SOA) is less than 40 ms, the prime (like a disk) is likely masked by and integrated with the target (a ring with inner diameter matching the diameter of the disk), leading to expanded duration of the target. However, when the SOA increases, the prime is consciously perceived and segmented from the target, resulting in duration compression. It appears that the perceptual organization between successive stimuli distorts the apparent duration of the stimulus. A similar illusion, in a form of chronostasis, can be demonstrated in situations where a voluntary action, such as a saccadic (Yarrow et al., 2001, 2004) or a manual (Park et al., 2003; Yarrow and Rothwell, 2003) movement, triggers the onset of a stimulus whose subjective duration is ultimately expanded relative to succeeding stimuli with the same physical durations. It is assumed that the illusory duration is substantiated by a mechanism to solve the uncertainty about the sensory onset caused by movement. However, other observations show the generalization of a chronostasis-like effect in audition and vision without voluntary action (Hodinott-Hill et al., 2002; Alexander et al., 2005; Hunt et al., 2008), indicating that motor action is neither necessary nor sufficient to produce chronostasis. Instead, more general functions such as arousal level, attention, and memory are proposed to account for the induced temporal dilation. Although these notions are intriguing, they may not cover the entire picture. A likely alternative is that both action-triggered and featural changes modulate the perceptual organization of the stimulus sequence that ultimately shape the object perception and the perceived duration of the target. Observers usually compare the relative duration of the target with that of other stimuli in the train, and it is arguable that the neural system segments the stimulus train and then estimates the target duration based on the average of comparison stimuli. Indeed, some studies have shown that visual temporal averaging (Ohyama and Watanabe, 2007) and auditory rhythmic grouping (Thorpe and Trehub, 1989; Geiser and Gabrieli, 2013) alter the temporal structure of stimulus patterns and lead to relatively longer subjective intervals between compared to within perceptual groups. Our own studies with double stimuli also reveal the role of segmentation and grouping of two visual events on the apparent duration of the second event (Zhou et al., 2010, 2014b). Interestingly, the subjective duration is modulated by the dominant grouping principle (Zhou et al., 2014b), thus manifests different patterns under various perceptual contexts.

It should be noted that the perceptual organization account does not conflict with other explanations of time distortion for brief intervals and durations. As a fundamental component of neural functions, perceptual organization profoundly interacts with other cognitive operations such as attention (Han et al., 2005; de Haan and Rorden, 2010), memory, and prior experience (Kimchi and Hadad, 2002; Zemel et al., 2002; Peterson and Berryhill, 2013). In a sense, perceptual organization under various influences sets the quality and content of our perceptual experiences (Herzog and Fahle, 2002; Wagemans et al., 2012), which may further serve as signals for the appreciation of elapsed time. On the basis of functional taxonomy (Pöppel, 1989), perceptual organization and temporal processing are logistical functions, whereas percepts represent content functions. Logistical functions usually modulate the way of contents being organized in content functions. There seems a loop that logistical functions influence content functions, which in turn carve the phenomenal representation of the former (Bao et al., 2013). Similar loop effects can be applied between logistical functions and other content functions, e.g., between the temporal organization and the content of memories. How does such loop implement itself using the “neural language”? A hypothesis advanced by Eagleman and Pariyadath (2009) may provide some clues; they argue that the consumed neural energy signals the length of the subjective duration, and neural repetition suppression is most probably underlying the time compression of repeated events. An oddball (Tse et al., 2004; Pariyadath and Eagleman, 2007, 2012; New and Scholl, 2009) or behaviorally salient stimulus (van Wassenhove et al., 2008; New and Scholl, 2009; Wittmann et al., 2010) can somehow escape the repetition suppression, thus leading to dilated apparent duration (relative to repeated stimuli). On the functional level, “oddball effects” may result from stimulus regularity and predictability (Pariyadath and Eagleman, 2007; Schindel et al., 2011) which nevertheless control the way of stimuli being perceptually organized. At what extent a stimulus can be segmented from other stimuli depends on its perceptual relation with others, on its ecological significance, or on its position in the stimulus stream. The finding that the duration of oddballs (Tse et al., 2004; Pariyadath and Eagleman, 2007, 2012), looming events (van Wassenhove et al., 2008; New and Scholl, 2009; Wittmann et al., 2010), and the first or last stimulus of a stream (Rose and Summers, 1995) is often overestimated may depend on their efficiency being separated from background events. The consequence of such process is the altered neural representation of these objects, and on the basis of the neural energy hypothesis (Eagleman and Pariyadath, 2009) their apparent durations. Similar within-sequence perceptual context and organization may also mediate the duration effect caused by speed changes even when the average speed is stable (Matthews, 2011a; Sasaki et al., 2013). Focusing on the relationship between subjective duration and perceptual organization highlights the importance of heuristic contexts and retrospective analyses on tracking events’ time. Thus, not only immediate contexts but also task environments can modulate the apparent duration of a stimulus (Pariyadath and Eagleman, 2007; Jazayeri and Shadlen, 2010; Matthews, 2011b; Zhou et al., 2014b).

As discussed above, perceptual organization modulates subjective duration via its role on shaping the representation of objects; it is natural to ask whether object features are also able to modify the perceived lapse of time. Answering this question, the apparent duration of a stimulus has been reported to link with its size (Xuan et al., 2007; Ono and Kitazawa, 2009; Alards-Tomalin et al., 2014), intensity (Nisly and Wasserman, 1989; Xuan et al., 2007), number of elements (Xuan et al., 2007), and spatial frequency (Aaen-Stockdale et al., 2011). Interestingly, psychological time is associated with perceived qualities and quantities of objects (Ono and Kawahara, 2007; Matthews et al., 2011; Yamamoto and Miura, 2012), rather than their physical properties. For example, a central circle appears to last longer when it is surrounded by smaller than by larger circles, although it has physically identical size and duration in both conditions (Ono and Kawahara, 2007). This effect is attributed to the different apparent sizes of the central circle induced by surrounding circles, a phenomenon termed Ebbinghaus illusion. In another study (Orgs et al., 2011), viewing an implied body, but not non-body or inverted-body, motion compresses subjective time. Interestingly, the compression effect depends on the apparent length of movement paths. Taken together with the observations in oddball and repetition paradigms, these findings strongly suggest that perceptual organization operating on both spatial and temporal domains influences how the target is temporally represented relative to other objects. This view exploits factors that shape the mental time from the point of perceptual functions and provides a useful supplement to other more biological concepts and hypotheses advocated previously (Eagleman and Pariyadath, 2009; Zhou et al., 2014a). Considering its dependence on neural signals encoding objects and their identities as well as its behavioral correlation with perceived object properties, subjective time can be treated as an object feature that is indirectly computed but nevertheless can be used to tag the object identity.

## Subjective Time Can Serve as a Research Tool to Examine Neural Representations of Objects

The association between the perceptual representation of objects and perceived time implies an important application of subjective time in the assessment of various mental processes. One can refer to the broad use of reaction time (RT) and its success in revealing characteristics of a variety of perceptual and cognitive operations since the beginning of experimental psychology (Pöppel, 1997; MacDonald and Meck, 2004; Posner, 2005). For a response to occur, accumulated neural signals, either detection or discrimination information, should reach a decision criterion which varies across trials but on average keeps stable for certain task sessions (Grice et al., 1982; Usher and McClelland, 2001). Usually, more strongly perceived objects lead to shorter RTs (Donner and Fagerholm, 2003; Palmer et al., 2005). Therefore, by inspecting RTs, one may obtain rich information about the neural codes of external inputs or the cognitive structures processing these inputs. In a similar vein, one can compare apparent durations of different objects under the same condition or of the same object under different conditions to study object representation and related neural processes. For example, Zhou et al. (2014b) presented observers two successive Gabor patches with varying orientation differences or spatial distances across trials. The apparent duration of the second Gabor patch is underestimated relative to its physical duration. Interestingly, the amount of duration compression is positively related to the size of orientation difference and spatial distance, in a way consistent with the orientation tuning and retinotopic mapping of early visual neurons. Similar results are also reported by Pariyadath and Eagleman (2012) when using an oddball paradigm. Thus, properties of the visual cortex can be inferred from the perceived duration of a visual stimulus without adaptation manipulation or neurophysiological recordings. The application of this concept can be found in another study (Aaen-Stockdale et al., 2011). In a typical oddball paradigm, observers compared the relative duration of an infrequent oddball (a Gabor patch) and that of a frequent standard which could be either another Gabor patch with different spatial frequency or an auditory stimulus. The authors found an interesting result that the duration expansion or compression of the oddball depended on its spatial frequency. Relative to lower and higher spatial frequencies, a mid-range spatial frequency (ca. 2 c/deg) consistently led to longer apparent duration that was invariant across different baseline durations. The perceived time in this example thus indicates that longer visual persistence is associated with neural responses selective to mid-range spatial frequencies in the early visual cortices. Other reports demonstrate spatially localized duration effects for drifting gratings and suggest neural sources in visual areas of striate and dorsal extrastriate cortices where neurons have specific receptive fields for moving objects (Johnston et al., 2006; Burr et al., 2007; Bruno et al., 2010). For more complex events, such as biologically relevant stimuli, studying apparent time also helps to uncover the neural processes involved in encoding these events (Wang and Jiang, 2012; Yamamoto and Miura, 2012). It has been found that merely observing the movement of another organism activates the mirror neuron system of the primate brain (Rizzolatti and Craighero, 2004). The question is whether a static image with implied motion also activates mirror neurons. Utilizing a duration judgment task, Yamamoto and Miura (2012) provide a positive answer that stationary images potentially dilate subjective time when they imply the running rather than the standing of a character. Such time effect suggests enhanced responses of mirror neurons induced by a static image with implied biological motion. In another study with point-light walker animation (Wang and Jiang, 2012), time expansion is associated with stimuli containing biological motion components, even when observers are unaware of their biological nature. Thus, apparent duration serves as a sensitive assessment of life-relevant signals and their neural substrates. However, one has to be cautious when employing subjective time as a tool. Various factors including stimulus features, contexts, and drug administration are known to influence time perception (Meck, 1996; Eagleman, 2008) and distributed brain areas are suggested to engage in the coding of subjective time (Mauk and Buonomano, 2004; Merchant et al., 2013). Therefore, it is a challenge to specify neural activities which are reflected in perceived time. This limitation calls for careful design of experiments to control possible confounding factors. Consulting well-established physiological and functional properties of certain neural structures may provide help in using perceived time as a research tool.

There is another advantage to measuring apparent time in psychological and neuroscience research. Object features and identities can be processed rapidly within the first tens to hundreds milliseconds after stimulus onsets (Thorpe et al., 1996). Thus, conventional RTs primarily reflect the state of transient neural responses caused by onsets. On the other hand, duration perception needs more information that can track the lapse of event. Not only onset transients, but also sustained responses and offset transients are involved in the temporal computation. Neurophysiological studies have argued that gradual changes of neural responses, e.g., ramping activities, over frontal and parietal areas serve the neural representation of mental time (see a review by Wittmann, 2013). Although such notion needs further elaboration to accommodate current doubts and inconsistencies, it is clear that certain sustained neural responses are forwarded as time signals which could be used for phenomenal representation. To this end, apparent time serves as a tool to assess, at least partially, the sustained neural responses between onset and offset transients. However, it is important to notice that there is currently no clear delineation between the contributions of transient and sustained signals to subjective time. Considering that properties of onset transients, mainly their amplitudes, profoundly alter the representation of brief time intervals (Noguchi and Kakigi, 2006; Terao et al., 2008), this challenge will significantly limit the application of subjective time in studies of sustained neural responses.

## Concluding Remarks

Perceptual organization is an essential component of the efficient coding of the world. Here, we highlight its role in modulating the temporal representation of objects and explore its biological consequences which might underlie experiences of brief time. As a way to structure the sensory inputs, perceptual organization modifies the neural responses as well as the homeostatic conditions induced by an object. Altered biological states may change the information that is transformed into a feeling of lasting long or short (Eagleman and Pariyadath, 2009; Wittmann, 2009; Zhou et al., 2014a). In this view, perceptual organization unifies a number of time illusions on a common principle and further enables us to predict the apparent duration in a given context. The retrospective and context-dependent nature also suggests that the link between perceptual organization and subjective time is applicable in time judgment not only for online objects but also for memorized events. Using subjective time as a research tool, on the other hand, complements other behavioral measurements such as RT paradigms. With this method, it is also possible to investigate both transient and sustained neural responses encoding object identity.

### Conflict of Interest Statement

The authors declare that the research was conducted in the absence of any commercial or financial relationships that could be construed as a potential conflict of interest.
